# Long non‐coding RNA VAL facilitates PKM2 enzymatic activity to promote glycolysis and malignancy of gastric cancer

**DOI:** 10.1002/ctm2.1088

**Published:** 2022-10-13

**Authors:** Ting Dai, Xin Zhang, Xiang Zhou, Xiaoxia Hu, Xiaodi Huang, Feiyue Xing, Han Tian, Yun Li

**Affiliations:** ^1^ Institute of Tissue Transplantation and Immunology, Department of Immunobiology Jinan University Guangzhou Guangdong China; ^2^ GMU‐GIBH Joint School of Life Sciences Guangzhou Medical University Guangzhou Guangdong China; ^3^ Clinical Experimental Center, Jiangmen Key Laboratory of Clinical Biobanks and Translational Research, Jiangmen Central Hospital Affiliated Jiangmen Hospital of Sun Yat‐sen University Jiangmen China; ^4^ Department of Microsurgery, Trauma and Hand Surgery The First Affiliated Hospital of Sun Yat‐sen University Guangzhou Guangdong China; ^5^ Zhongshan School of Medicine Sun Yat‐sen University Guangzhou Guangdong China

**Keywords:** gastric cancer, glycolysis, Parkin, PKM2, VAL

## Abstract

**Background:**

Gastric cancer (GC) is one of the most common types of cancer worldwide, which leads to more than 10% of cancer‐related deaths. Metabolism reprogramming presents as a pivotal event in cancer initiation and progression through enhancing aerobic glycolysis and anabolic metabolism. However, the underlying regulatory mechanisms in GC remain unknown.

**Methods:**

VAL was identified by bioinformatics analyses in GC. Cell‐based assays and mouse model illustrate the role of VAL in GC. RNA pull‐down, immunoprecipitation assay and Western blot elucidate the interaction between VAL and PKM2. Pyruvate kinase activity, ECAR and OCR were measured to validate aerobic glycolysis of GC cells.

**Results:**

Long non‐coding RNA (lncRNA) VAL is significantly upregulated in GCs and indicates poor prognosis. Functional assays showed that VAL promotes GC malignant progression. Mechanistically, VAL strengthens the enzymatic activity of PKM2 and aerobic glycolysis of GC cells through directly binding with PKM2 to abrogate the PKM2–Parkin interaction, and to suppress Parkin‐induced polyubiquitination of PKM2. In addition, glucose starvation induces VAL expression to enhance this process.

**Conclusions:**

Our study provides an insight into an lncRNA‐dependent regulation on the enzymatic activity of PKM2, and suggests a potential of targeting VAL or PKM2 as promising biomarkers in GC diagnosis and treatment.

## Graphical abstract

VAL functions as an important regulator of PKM2‐dependent glycolysis process and a propellant of gastric cancer (GC) progression.

PKM2 enhances PKM2 enzymatic activity by abrogating Parkin‐mediated polyubiquitination.

High VAL expression correlates with poor prognoses of GC patients, suggesting that VAL is a promising prognostic and therapeutic marker for GCs.

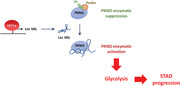

## 1 BACKGROUND

Gastric cancer (GC) is one of the most lethal malignant cancers worldwide, the morbidity and mortality of it ranks top 5 among all cancer types.^1^ Notably, approximately half of newly diagnosed GC cases globally are found in China.^2^ Despite the great progress in GC diagnosis and treatment over the past decades, including improved surgical procedures and up‐to‐date targeting therapy, the prognosis of patients with GC worldwide, especially in China, remains suboptimal.^3^, ^4^ Rapid progression with imperceptible symptoms and potently invasive and metastatic ability of GC is the leading cause of the poor prognosis and cancer‐related death of GC patients.^5^ Therefore, elucidating the underlying mechanisms of GC malignant progression has been well accepted as a pressing need in this area, which may lead to discovery of effective prognostic/therapeutic markers for GC.

Reprogramming of cellular metabolism has been well recognized in cancers, which fuels growth and division of malignant transformed cell, and leads to oncogene activation and conferring malignant phenotypes on cancer cells.^6^ Markedly increased uptake and utilization of nutrients or biomass, especially glucose, and dysregulated expressions and enzymatic activities of metabolic enzymes have been well documented in various types of cancer cells.^7^ At the transcriptional level, oncogenic transcriptional factors c‐Myc and HIF1α induce multiple glycolytic enzymes expression, such as GLUTs, HKs, LDHA and PKM2, rendering metabolic reprogramming of cancer cells.^8^, ^9^ In addition, post‐transcriptional modifications and non‐coding RNAs also function as key regulators of the enzymatic activity, subcellular localization and RNA/protein stability of the glycolytic enzymes.^10^, ^11^, ^12^, ^13^ Significantly, the Warburg effect, a phenomenon in cancer cells that the main metabolic pathway of glucose switches from oxidative phosphorylation (OXPHOS) to glycolysis, with production of massive biomass for cell growth, presents as a fundamental feature of cancer cells.^14^ Among the pathway, PKM2, a critical enzyme that is responsible for the last step of glycolysis, has been considered as a promising therapeutic target for cancer, which converts phosphoenolpyruvate (PEP) to pyruvate with production of ATP, fueling the tricarboxylic acid (TCA) cycle.^15^, ^16^ Although the mechanisms of elevated PKM2 enzymatic activity or expression level in multiple types of cancers have been widely investigated,^17^ the veiled molecules that promote cancer malignant progression via PKM2‐mediated glycolysis in GC remain poorly understood.

Long non‐coding RNA (lncRNA) is a kind of emerging RNA molecule that is involved in multiple pathways to regulate cellular biological processes relying on the interaction with functional molecules.^18^, ^19^ Accumulated studies show that lncRNAs, such as HOTAIR, NKILA, TINCR and PVT1, promote cancer cell motility, immortality and proliferation in diverse molecular mechanisms, including chromatin‐, protein‐ and RNA‐interaction dependent manners.^20^, ^21^, ^22^, ^23^ Moreover, lncRNAs have been identified as promising biomarkers for multiple cancer types, like breast cancer, hepatocellular cancer, prostate cancer and gastric cancer.^20^, ^24^, ^25^, ^26^ Notably, accumulated studies also illustrated that lncRNAs participate in the regulation of glycolysis in cancer cells, such as glycoLINC, HIFAL, DLEU2, WFDC21P, LINC01123 and LINC00538, which enhance HIF1α or c‐Myc‐mediated transcriptome, modulate glycolytic flux and stability or activity of glycolytic enzymes at different levels.^27^, ^28^, ^29^, ^30^, ^31^, ^32^ In this light, our knowledge about the pathophysiological role of lncRNAs in glycolysis and malignancy regulation of GC remains elusive.

Here, we report that our previously identified lncRNA VAL is markedly elevated in GCs and predicts poor prognosis of GC patients. In addition, cell‐ and mouse‐based gain‐ or loss‐of‐function assays reveal that VAL plays a pivotal role to promote malignant progression of GC, including proliferation, invasion and chemoresistance. We further prove that VAL facilitates PKM2 enzymatic activity and aerobic glycolysis through directly binding to PKM2 and suppressing the polyubiquitination of PKM2 by abrogation of the PKM2–Parkin interaction. Additionally, glucose starvation elevates VAL levels to facilitate this process. Our study provides an insight into the regulation of PKM2 at the post‐translational level and opportunities for GC diagnosis and treatment.

## 2 METHODS

### 2.1 Cell lines

AGS, MGC803, SNU16, NCI‐N87, MKN28, MKN45 and KATOIII gastric cancer cell lines, GES‐1 normal gastric epithelial cell line, and HEK293FT human embryonal kidney cell were purchased from National Collection of Authenticated Cell Cultures (NCACC). Cell lines were cultured in indicated mediums supplemented with 10% fetal bovine serum (Gibco): DMEM for HEK293FT cell line, RPMI‐1640 for GES‐1, MGC803, SNU16, NCI‐N87, MKN28, MKN45 and KATOIII cell lines and Ham's F‐12K (Kaighn's) Medium for AGS cell line. Aforementioned cell lines were all authenticated by short tandem repeat (STR) analysis.

### 2.2 Clinical specimens

The GC cohort used in our study was collected from Jiangmen Central Hospital, which contains 116 GC tissues and five gastric benign hyperplasia tissues. Our study is compliant with all ethical stipulates. Study approval and patients’ consents were obtained from the Institutional Research Ethics Committee of Jiangmen Central Hospital.

### 2.3 Animal model

In our study, male BALB/c‐nude mice (18–20 g) were fed in specific pathogen‐free (SPF) facilities. For xenograft assays, 100 μl of GC cells with concentration at 1 × 10^7^ cells/ml were subcutaneously inoculated into the flanks of mice. Compound 3K dissolved in specific solvent (10% DMSO and 90% corn oil) was administered orally every 2 days (5 mg/kg), and the specific solvent was administered orally as control. Mice were sacrificed at 5 weeks after inoculating GC cells. Bidimensional measurements of xenograft tumours were collected by using Vernier calipers and the volume of tumours was calculated according to the formula: *V* (mm^3^) = (*L* × *W* ^2^)/2. The mouse model experiments in our study were approved by the Institutional Animal Care and Use Committee of Jinan University.

### 2.4 TCGA STAD dataset

RNA expression data of adjacent normal tissues and GC tissues, and their corresponding pathological and prognosis material, were downloaded from The Cancer Genome Atlas Stomach Adenocarcinoma (TCGA STAD) cohort.

### 2.5 Plasmids, virus production and transfection

VAL (LINC01546) was cloned into pSin‐EF2‐puro retroviral vector (Addgene), and Flag‐PKM2 and HA‐ubiquitin were separately cloned into pCDNA3.4 TOPO vector (Invitrogen). For depletion of VAL (targeting sequence, sh1: 5′‐GCACAATAGGCACAAGAATGA‐3′, sh2: 5′‐GCTATTCACCATTTGGTTTCT‐3′) or Parkin (targeting sequence, sh1: 5′‐GCACACCCCACCTCTGACAAGG‐3′, sh2: 5′‐GCTTTTATGTGTATTGCAAAGG‐3′), human shRNA sequences against each of them were, respectively, cloned into the pSuper‐retro‐neo vectors (OligoEngine). For construction of stable cell lines overexpressing VAL or knocked down for VAL, pSin‐VAL plasmid was co‐transfected with pMD2G and psPAX2 packaging plasmids, and plasmid carrying distinct VAL shRNAs were co‐transfected with PIK plasmid into HEK293FT cell by Lipofectamine 3000 reagent (Invitrogen); subsequently, cell culture supernatants of HEK293FT cells were collected to infect GC cells for 24 h with polybrene (8 μg/ml), followed by selection with corresponding antibiotics for 1–2 weeks. In addition, Lipofectamine 3000 reagent was employed for transient transfection of plasmids.

### 2.6 Antibodies and reagents

Primary antibodies were: anti‐FLAG (F1804, Sigma‐Aldrich), anti‐HA (51064‐2‐AP, Proteintech), anti‐PKM2 (D78A4, CST) and anti‐Parkin (66674‐1‐Ig, Proteintech), and normal mouse IgG for immunoprecipitation (12‐371, Sigma‐Aldrich). Blotted PVDF membranes were re‐blotted with anti‐GAPDH antibody (10494‐1‐AP, Proteintech) used as loading controls. Protease Inhibitor Cocktail (C0001) was purchased from TargetMol. PKM2 inhibitor Compound 3K (S8616), 5‐fluorouracil (5‐FU) (S1209) and oxaliplatin (S1224) were purchased from Selleck.

### 2.7 RNA extraction and qRT‐PCR

Total RNAs from surgically resected fresh GC tissues or cultured cell lines were prepared by using TRIzol reagent (Invitrogen). MMLV transcriptase (Promega) with random primers was used for generating cDNA by reverse transcription. CFX96 real‐time PCR platform (Bio‐Rad) was employed for qRT‐PCR assays. Relative gene expressions were calculated as follow: 2^−[(Ct of mRNA) – (Ct of GAPDH)]^. The primers used in our assays are VAL forward, 5′‐TAGGCACAAGAATGAACCAA‐3′; VAL reverse, 5′‐GGAATACCGATGAAGCAGAT‐3′; GAPDH forward, 5′‐GACTCATGACCACAGTCCATGC‐3′; GAPDH reverse, 5′‐AGAGGCAGGGATGATGTTCTG‐3′.

### 2.8 Cell Counting Kit‐8 assay

Following the manufacturer's instruction (CK04, Dojindo), indicated cells (1000 cells/well) were seeded into 96‐well plates in triplicate. Cell viability of indicated cells was measured for 6 days after seeding. Before detection, 10 μl of Cell Counting Kit‐8 (CCK‐8) solution was added and incubated for 2 h, then the optical density (OD) values of each well were determined at 450 nm.

### 2.9 Transwell assays

Indicated cells were seeded into the transwell chamber (Millipore) with precoated Matrigel (BD Biosciences). After 24 h, cells that had invaded into or through the membrane were fixed and stained with 0.1% crystal violet. Cells in randomly selected microscopic fields of each well were counted.

### 2.10 Analysis of apoptosis cells

Indicated cells were digested into single cell status and stained with propidium iodide (PI; 10 μg/ml; Sigma‐Aldrich) and Annexin V‐FITC (50 μg/ml, BD Biosciences) for 15 min, then the cells were analyzed by flow cytometry.

### 2.11 Endogenous RNA pull‐down assay with mass spectrometry analysis

For endogenous RNA pull‐down assay, biotinylated short probes against VAL or its antisense (used as negative control) were designed and synthesized as described in the single‐molecule RNA fluorescence in situ hybridization (smFISH) assay. According to the Chang lab's protocol, the cell pellets were prepared and re‐suspended in specific lysis buffer on ice for 10 min, then the lysis was sonicated using Bioruptor (Diagenode) for 2 min at 50 W in ice bath and diluted in two times volume of hybridization buffer, followed by addition of 100 pmol biotinylated probes for hybridization by rotation at 37°C for 4 h. Dynabeads Streptavidin C1 beads (Sigma‐Aldrich) were incubated with the whole above mixture and captured by magnets. The beads biotinylated probes‐RNAs‐proteins adducts were washed five times with wash buffer, resuspended in DNase buffer and eluted for bound proteins with elution buffer. At last, eluted proteins were analyzed by SDS‐PAGE with manifested silver staining. Distinct bands of both VAL‐interacting or VAL antisense‐interacting proteins located at the same molecular weight in the gels were separately cut and subjected to mass spectrometry (MS) analysis (Applied Protein Technology). Notably, although the endogenous RNA pull‐down assays for identifying VAL‐interacting proteins were repeated three times, which similarly revealed distinct bands as manifested by silver staining, MS analysis of both VAL‐interacting or VAL antisense‐interacting proteins was done without biological or technical replicates. The proteins identified by MS analysis were further confirmed by endogenous RNA pull‐down and Western blots assays.

### 2.12 Co‐immunoprecipitation and RNA immunoprecipitation

Indicated cells were washed with PBS and then were lysed by lysis buffer supplemented with 0.2% Cocktail (Promega), then the cell lysate was incubated with 50 μl anti‐Flag agarose beads or anti‐mouse IgG agarose beads overnight at 4°C. The beads were then washed five times with wash buffer and subjected to WB analysis. RNA immunoprecipitation (RIP) analysis was performed by using Magna RIP RNA‐Binding Protein Immunoprecipitation Kit (Millipore). Briefly, cells cultured on dishes were UV crosslinked, then lysed in RIP lysis buffer supplemented with 0.2% Cocktail and 0.1% RNase inhibitor, and subjected to immunoprecipitation. The immunoprecipitated complex was digested with proteinase K, followed by RNA extraction and quantitation of VAL using qRT‐PCR.

### 2.13 Single‐molecule RNA fluorescence in situ hybridization

Staining of VAL in cultured cells was performed by smFISH according to the Sanjay Tyagi lab's procedures. Short probes labeled with Alexa Fluor 647 in 3′ ends were designed in Stellaris‐Probe‐Designer online program and synthesized by Invitrogen. Cells seeded on cover‐slips were briefly washed with PBS, and fixed with formaldehyde, then permeabilized in 70% ethanol for 1 h at 4°C and washed in hybridization buffer for 5 min. Subsequently, hybridization was carried out using anti‐VAL oligonucleotide probe sets for 4 h at 37°C in a moist chamber, followed by washing of the cells for 30 min at 37°C in wash buffer. After the hybridization, cells on cover‐slips were counterstained with DAPI, and representative images were obtained with a LSM810 confocal microscope using ZEN 2012 software version 8.1 (Carl Zeiss).

### 2.14 Pyruvate kinase activity assay

Purified PKM2 or ubiquitinated PKM2 protein was immunoprecipitated and eluted by elution buffer. Next, the purified protein was added to reaction buffer, and PKM2 activity was measured with a PK activity assay kit (709‐100, BioVision).

### 2.15 Measurement of glucose consumption, lactate production, ATP production, OCR and ECAR

Glucose Uptake Colorimetric Assay Kits (Biovision), Lactate Colorimetric Assay Kits (Biovision) and ATP Assay Kit (S0026, Beyotime) were used according to the manufacturer's instructions. The Seahorse Bioscience XFe96 Extracellular Flux Analyzer platform (Seahorse Bioscience) was employed to analyze the mitochondrial oxygen consumption rate (OCR) and extracellular acidification rate (ECAR). Briefly, 2 × 10^4^ cells were seeded per well in the XF96 plate and incubated overnight. Rinsing the cell with Seahorse buffer, 175 μl of Seahorse buffer with 25 μl each of oligomycin (1 μM), FCCP (1 μM) or rotenone (1 μM) were automatically added to measure the OCR. And 25 μl each of glucose (10 mM), oligomycin (1 μM) or 2‐deoxy‐glucose (100 mM) were added to measure the ECAR. The results were analyzed by using the Seahorse software.

### 2.16 Statistical analysis

Statistical analyses were performed by using GraphPad Prism 8 software (GraphPad Software, San Diego, CA, USA). Comparisons between two groups were performed using Student's *t*‐test (two‐tailed), and analyses between multiple treatments were performed using two‐way ANOVA. Survival curves were analyzed by the Kaplan–Meier method with log‐rank test. All error bars represented the mean ± SD derived from three independent experiments. In all cases, *p* < .05 was considered to be statistically significant; *p < .05, **p < .01.

## 3 RESULTS

### 3.1 Upregulated VAL expression predicts poor prognosis of gastric cancer patients

**FIGURE 1:**
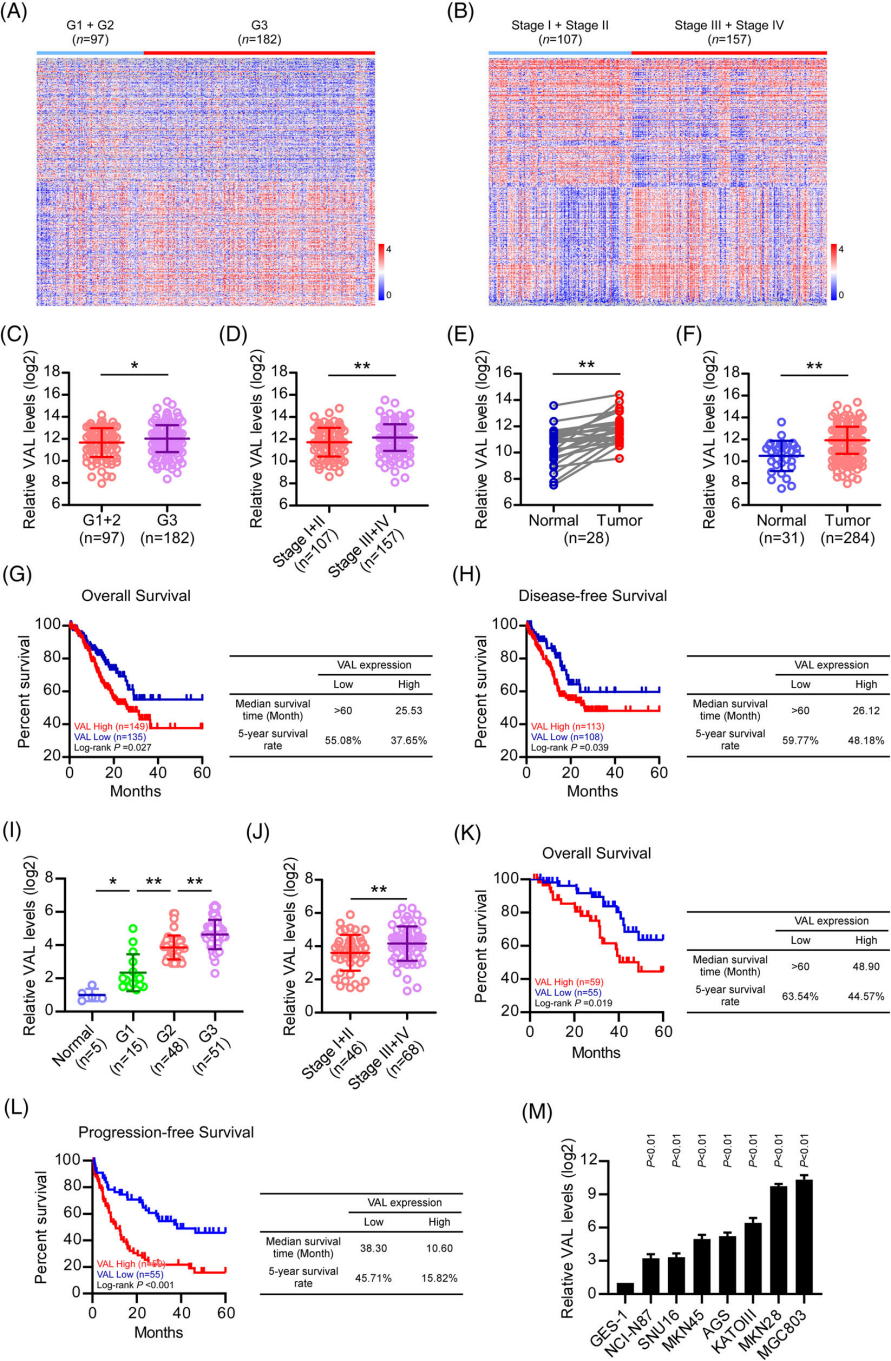
Upregulated VAL expression predicts poor prognosis of gastric cancer patients. (A and B) Heatmap shows the genes significantly altered in stomach adenocarcinoma tissues with higher histological grade or TNM stage of the TCGA STAD dataset. (C–F) Analysis of VAL expressions in histological grade 1–3 GC tissues (C), in TNM stage I–IV GC tissues (D), in 28 pairs of human GC tissue and adjacent normal tissue (E) and in 31 cases of adjacent normal tissues and 284 cases of GC tissues (F) of the TCGA STAD dataset. (G and H) Kaplan–Meier analysis (log‐rank test) of the 5‐year overall survival (G) and disease‐free survival (H) of GC patients in the TCGA STAD cohort, who were divided into low‐ or high‐VAL expression subgroups. (I and J) Analysis of VAL expression in benign gastric hyperplasia and histological grade 1–3 GC tissues (I), or in TNM stage I–IV GC tissues (J) of our collected cohort. (K and L) Kaplan–Meier analysis (log‐rank test) of the 5‐year overall survival (K) and progression‐free survival (L) of GC patients in our collected cohort. (M) Analysis of VAL expression in human gastric mucosal epithelial cell line GES‐1 and series of human GC cell lines. Results are presented as mean ± SD of at least three independent experiments. *p < .05, **p < .01

To identify the key molecules associated with GC progression, we analyzed the profiles of the TCGA STAD dataset using classifications of clinicopathological characters, including pathological grade and TNM stage (Figure 1A,B). Among the significantly dysregulated genes, we found that the lncRNA VAL (vimentin‐associated lncRNA, LINC01546, CXorf28) was markedly upregulated in both third histological grade, and III/IV TNM stage patients as compared to patients bearing lower grade/stage GCs (Figure 1C,D, Figure S1A and Tables S1 and S2). VAL has been reported as a pro‐metastatic molecule, which is upregulated by aberrant AKT signaling in lung adenocarcinoma (LAD) and promotes LAD metastasis, suggesting that VAL is a promising marker for diagnosis and treatment of GCs.^33^ Therefore, to understand the potential role of VAL in GC malignant progression, the expression of VAL was further examined in TCGA STAD dataset, and found that VAL was significantly upregulated in GC tissues, comparing with adjacent normal tissues, which contains 284 gastric cancer tissues and 31 normal tissues, including 28 pairs of cancer and adjacent normal tissues (Figure 1E,F). In addition, another published Gene Expression Omnibus (GEO) dataset also illustrated that VAL was markedly elevated in GCs as compared to adjacent normals in Chinese cohort (Figure S1B). Moreover, Kaplan–Meier plot survival analysis of the overall survival (OS) showed that GC patients with higher VAL levels were positively correlated with a significantly poor prognosis, and that the median survival time was 25.53 months and the 5‐year survival rate was 37.65%, as compared to the patients with lower VAL levels, that median survival time was >60 months and the 5‐year survival rate was 55.08% (Figure 1G). Meanwhile, VAL expression level was also associated with shorter disease‐free survival (DFS), that the median survival was 26.12 months and the 5‐year survival rate was 48.18% in the VAL high subgroup versus the median survival was >60 months and the 5‐year survival rate was 59.77% in the VAL low subgroup of TCGA STAD cohort (Figure 1H).

In light of the clinical significance of VAL in TCGA STAD cohort, we further assessed the clinical significance of VAL expression in the Jiangmen cohort, which contains 114 GC specimens and five benign hyperplasia specimens. Analogously, VAL expression levels in GCs were markedly elevated as compared with that in benign hyperplasia tissues and were increased accompanied with tumour progression (histological grade and TNM stage) in GCs (Figure 1I,J). In accordance with the mentioned results, Kaplan–Meier plot survival analysis in the same cohort also revealed that GC patients with higher VAL expression was correlated with a significantly shorter OS time and progression‐free survival time (OS: median survival 48.90 months and 44.57% 5‐year survival rate of VAL high subgroup vs. median survival >60 months and 63.54% 5‐year survival rate of VAL low subgroup; progression‐free survival: median survival 10.60 months and 15.82% 5‐year survival rate of VAL high subgroup vs. median survival 38.30 months and 45.71% 5‐year survival rate of VAL low subgroup) (Figure 1K,L). In addition, the analyses between VAL levels and clinicopathological characters of each GC patient showed a significant correlations between higher VAL expression and histological grade, TNM stage, T classification and N classification (Table 1). Moreover, consistent with other clinicopathological characters, VAL was proved as an independent risk factor for poor prognosis of GC patients in the Jiangmen cohort (Table 2).

**TABLE 1:** Relationship between the expression levels of VAL and clinicopathological characteristics of gastric cancer patients

		**VAL**				
**Characteristics**		**Low (*n* = 55)**	**High (*n* = 59)**	**Total**	***χ^2^***	***p***
Age	<60	28 (50.0%)	28 (50.0%)	56	0.136	.713
	≥60	27 (46.6%)	31 (53.4%)	58		
Gender	Female	18 (45.0%)	22 (55.0%)	40	0.260	.610
	Male	37 (50.0%)	37 (50.0%)	74		
Histological grade	G1	13 (86.7%)	2 (13.3%)	15	26.630	<.001
	G2	30 (63.8%)	17 (36.2%)	47		
	G3	12 (23.1%)	40 (76.9%)	52		
TNM stage	I+II	27 (58.7%)	19 (41.3%)	46	3.373	.066
	III+IV	28 (41.2%)	40 (58.8%)	68		
Tumour size	T1+T2	13 (68.4%)	6 (31.6%)	19	6.929	.031
	T3	34 (50.0%)	34 (50.0%)	68		
	T4	8 (29.6%)	19 (70.4%)	27		
LN metastasis	N = 0	24 (66.7%)	12 (33.3%)	36	7.151	.008
	N > 0	31 (39.7)	47 (60.3%)	78		
Distant metastasis	M = 0	51 (51.0%)	49 (49.0%)	100	2.474	.116
	M > 0	4 (28.6%)	10 (71.4%)	14		

**TABLE 2:** Multivariate analysis of prognostic factors for survival in patients with gastric cancer

**Characteristics**	**Progression‐free survival**			**Overall survival**		
	***p***	**HR**	**95% CI**	***p***	**HR**	**95% CI**
Histological grade (G1/G2+G3)	.004	0.176	0.055–0.566	.053	0.241	0.057–1.020
TNM stage (Ⅰ+Ⅱ/III+IV)	.000	0.324	0.191–0.549	.005	0.207	0.068–0.624
Tumour size (T1+T2/T3+T4)	.045	0.370	0.141–0.976	.086	0.596	0.203–1.751
LN metastasis (N0/N > 0)	.013	0.443	0.233–0.845	.008	0.152	0.038–0.615
Distant metastasis (M0/M > 0)	.009	0.406	0.204–0.808	.043	0.265	0.129–0.555
VAL expression (lower/higher)	.003	0.487	0.301–0.788	.034	0.456	0.221–0.941

Furthermore, we measured VAL levels in multiple GC cell lines and normal gastric epithelial cell line GES‐1. As expected, VAL RNA levels were elevated to varying degrees in GC cell lines than that in normal cell (Figure 1M). Taken together, our analysis demonstrated that VAL expression is elevated in GC with cancer progression and positively associates with poor prognosis of GCs.

### 3.2 VAL promotes malignant progression of gastric cancer

**FIGURE 2:**
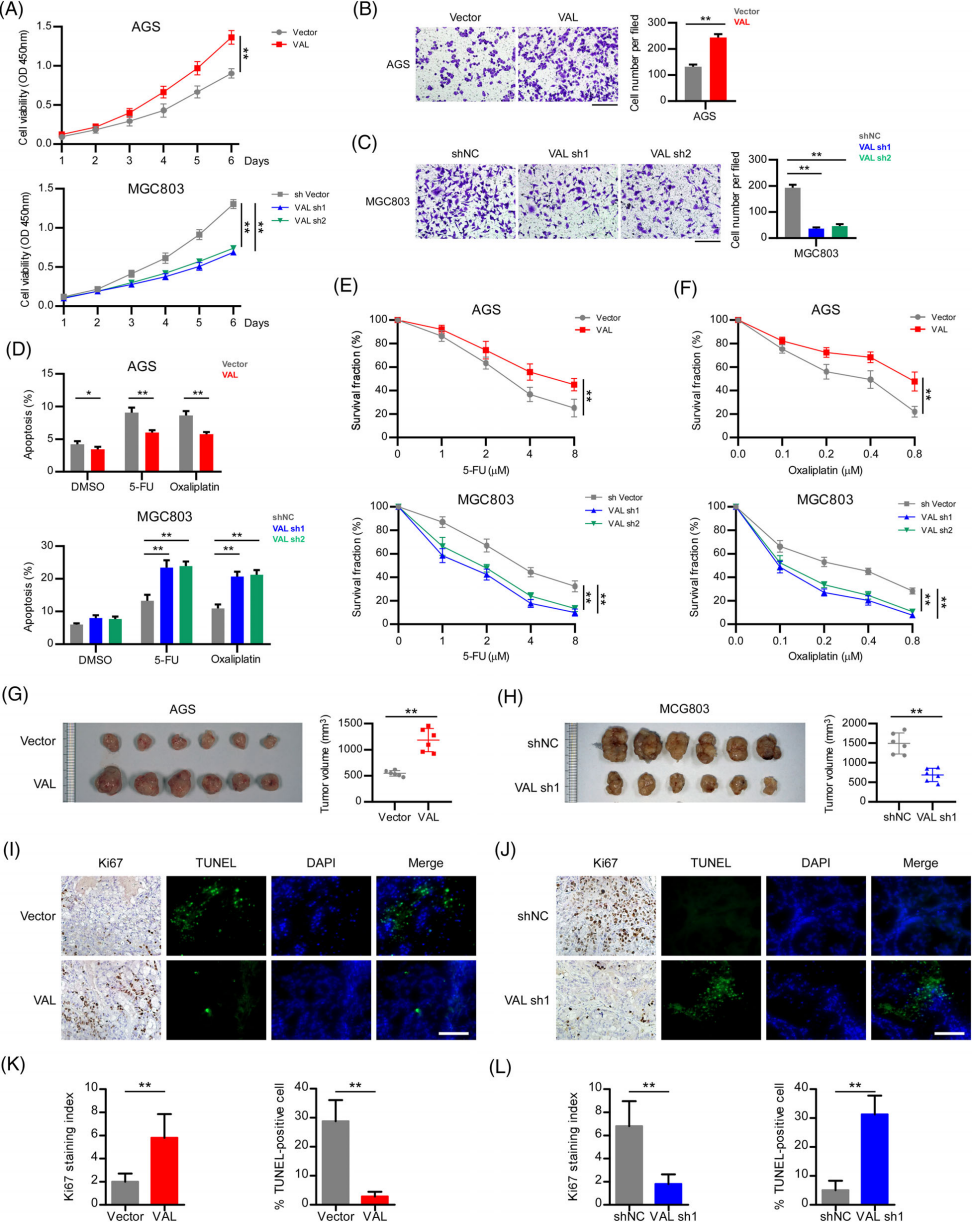
VAL promotes malignant progression of gastric cancer. (A) CCK8 assays were conducted in the indicated cells. (B and C) Representative images (left) and quantification (right) of the indicated AGS (B) and MGC803 (C) cells cultured for 24 h on Matrigel‐coated plates (five random fields of view per well, scale bar: 100 μm). (D) Apoptosis rates were measured by FACS analysis in the indicated cells treated with 10 μM 5‐FU or 5 μM oxaliplatin for 24 h. (E and F) CCK8 assays were conducted in the indicated cells treated with the indicated concentrations of 5‐FU (E) or oxaliplatin (F). (G–L) Images of gross specimen, Ki67 staining and TUNEL staining of subcutaneous xenograft dissected from nude mice inoculated the indicated AGS (G and I) and MGC803 (H and J) cells, and the volume of subcutaneous xenograft in each group (right panel of G and H), and staining index of indicated assays (K and L) (scale bar: 100 μm). Results are presented as mean ± SD of at least three independent experiments. *p < .05, **p < .01

To understand the role of VAL in GC progression, three GC cell lines with low, medium or high expression levels of VAL were selected for gain‐ or loss‐of‐function experiments. Specifically, we overexpressed VAL in AGS and SNU16 cells, and silenced VAL in MGC803 cells (Figure S2A,B). As compared to vector‐control cells, overexpression of VAL significantly enhanced the abilities of cell proliferation and invasion in AGS and SNU16 cells (Figure 2A,B and Figure S3A–D). By contrast, knockdown of VAL markedly impaired proliferation and invasion abilities of MGC803 cells (Figure 2A,C and Figure S3B,C). In addition, when cultured cells were treated with 5‐FU or oxaliplatin, GC cells with VAL overexpression showed enhanced resistance to chemotherapeutic drug‐induced apoptosis and retained cell growth, whereas silencing of VAL greatly promoted cell apoptosis and suppressed cell growth (Figure 2D–F and Figure S2E–G). These results suggested that upregulated VAL contributes to malignant phenotype of GC cells, including proliferation, invasion and chemoresistance.

In accordance with the in vitro assays, subcutaneous xenograft nude mice models showed that the VAL‐overexpressed AGS cell formed tumours with significantly larger volumes, higher Ki67 staining index and less TUNEL‐positive cells, as compared with the vector cells formed tumours (Figure 2G,I,K). Conversely, knockdown of VAL markedly reduced the ability of subcutaneous tumour formation of MGC803 cell with diminished cell proliferation and enhanced apoptosis (Figure 2H,J,L). In addition, comparing with the AGS‐vector cell, VAL‐overexpressed AGS cell showed potently invasive ability to intrude into the adjacent subcutaneous tissue and formed irregularly invasive front (Figure S3H); whereas, silencing of VAL greatly diminished the invasive ability of MGC803 cell, which generated subcutaneous tumours with smooth tissue boundary (Figure S3I). Taken together, these findings illustrate that VAL is crucial for malignant progression of GC.

### 3.3 VAL interacts with PKM2 in gastric cancer cells

To further understand the underlying mechanism of VAL promoting GC progression, first, the biological processes associated with VAL expression level were identified by the Metascape program (metascape.org), which showed that the significant altered genes between two subgroups defined by the upper and lower quartile of VAL expression level in TCGA STAD specimens (Figure S4A) were correlated with the metabolic process as the top‐level enriched gene ontology process and the ATP metabolic process as the most significantly enriched term (Figure S4B,C), suggesting that VAL is involved in regulation of GC cell metabolism.

**FIGURE 3:**
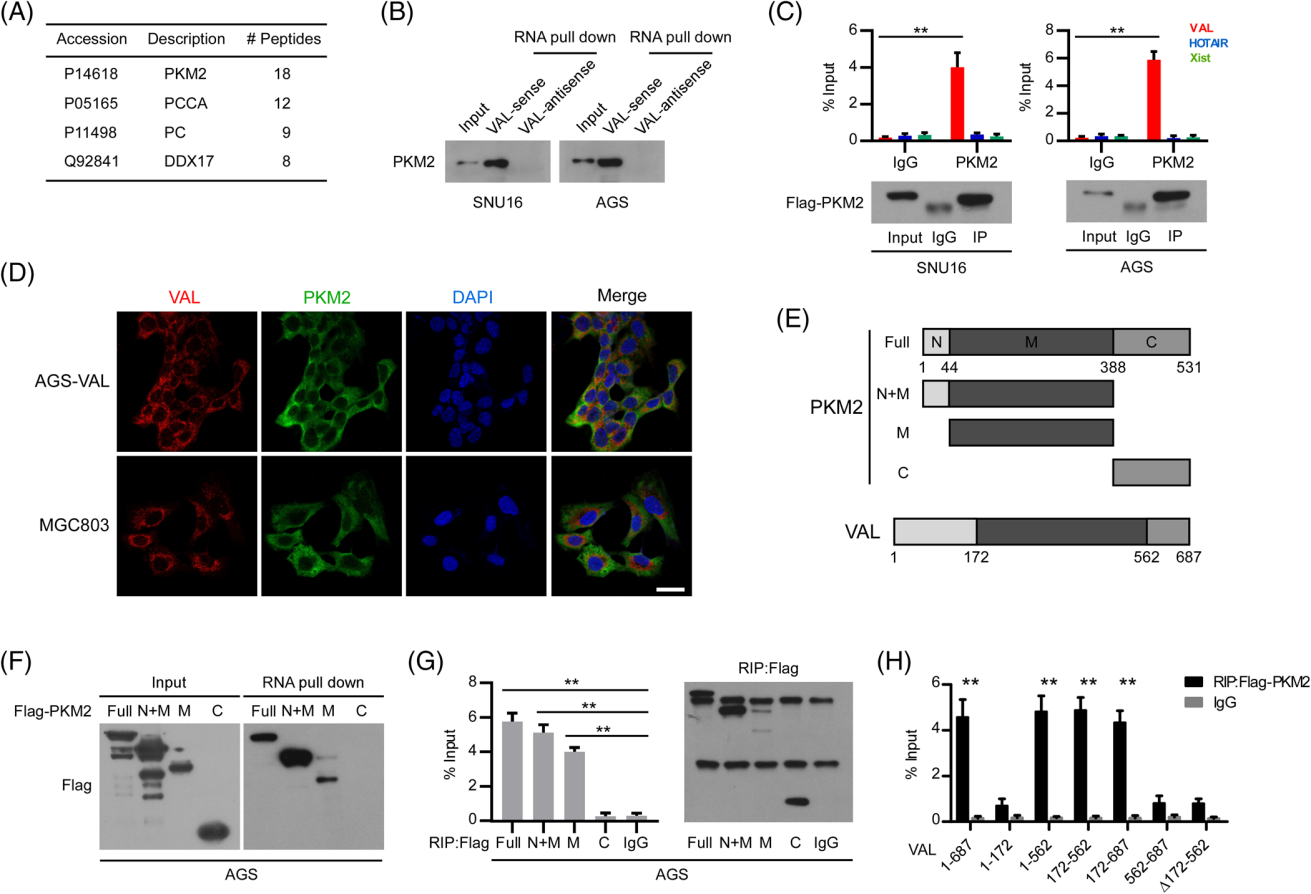
VAL interacts with PKM2. (A) Proteins bound to VAL in AGS cells were separated by SDS‐PAGE following endogenous RNA pull‐down assay, and analysis results of the eluted proteins by mass spectrometry are shown. (B) Endogenous RNA pull‐down assays validate the specific interaction between VAL and PKM2 in the indicated cells. Antisense RNA of VAL was used as negative controls. (C) RIP assays showed the interaction of PKM2 with VAL, but not with HOTAIR or Xist. (D) Representative images of co‐staining of VAL and PKM2 in the indicated cells using smFISH and immunostaining assays together (five random fields of view per slice, DAPI: 4′,6‐diamidino‐2‐phenylindole, scale bar: 35 μm). (E) Schematic diagram of the PKM2 protein domains and VAL truncations. (F) Exogenous RNA pull‐down assays showed the interaction between VAL and different truncated mutants of PKM2. (G and H) RIP assays showed the interaction between truncated mutants of Flag‐tagged PKM2 and VAL. Representative images of three independent reproducible experiments are presented (B, C, F, G and H). Results are presented as mean ± SD of at least three independent experiments. **p < .01

Subsequently, endogenous RNA pull‐down assays were conducted in AGS cell with transient expression of VAL as previously described.^33^ Following MS analysis it was found that PKM2, a pivotal protein in glycolysis, is abundantly enriched by VAL and is considered as a potential binding protein of VAL (Figure 3A), and the interaction was further confirmed in SNU16 and AGS cells by RNA pull‐down and Western blot assays (Figure 3B). In parallel, the specific interaction between Flag‐tagged PKM2 and VAL was validated by RIP assays (Figure 3C). Moreover, smFISH with immunofluorescence staining and qRT‐PCR with subcellular fractions showed that both endogenous and ectopic overexpressed VAL were colocalized with PKM2 in the cytoplasm of GC cells (Figure 3D and Figure S4D).

To further understand their interaction, PKM2 and VAL truncations were constructed to identify the regions that are responsible for their interaction (Figure 3E). Following, AGS cell transfected with Flag‐tagged PKM2 truncations were subjected to RNA pull‐down assays, which revealed that the N‐terminal and the middle domain of PKM2 were responsible for interacting with VAL (Figure 3F). Moreover, consistent results were obtained by RIP assays of Flag‐tagged PKM2 truncations (Figure 3G). As for VAL, RIP assays of Flag‐tagged PKM2 with VAL truncations suggested the 172–562 nt of VAL bond PKM2 (Figure 3H). Taken together, these results illustrate that VAL is a novel binding partner of PKM2 in GC cells.

### 3.4 VAL abrogates the interaction between PKM2 and Parkin

**FIGURE 4:**
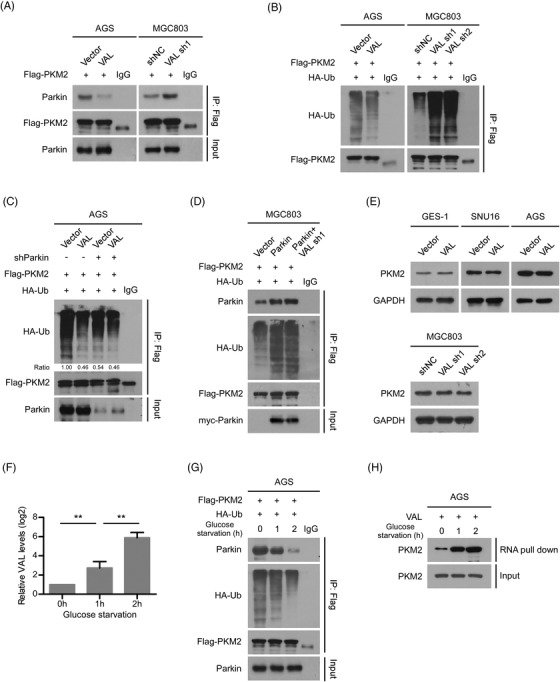
VAL abrogates the interaction between PKM2 and Parkin. (A) The effect of VAL on the interaction between Parkin and PKM2 was evaluated by immunoprecipitation of Flag‐tagged PKM2 in the indicated cells. (B) The effect of VAL on the levels of polyubiquitination of PKM2 was evaluated by immunoprecipitation of FLAG in the indicated cells. (C) The effect of silencing Parkin on the levels of polyubiquitination of PKM2 in the indicated cells. (D) The effect of overexpressing Parkin or silencing VAL with overexpressing Parkin on the levels of polyubiquitination of PKM2 in the indicated cells. (E) WB analysis of PKM2 expression in the indicated cells. (F) Relative RNA levels of VAL of indicated cells after glucose starvation for 0, 1 and 2 h, respectively. (G) Co‐IP assay showed the interaction between Parkin and PKM2, and the levels of polyubiquitination of PKM2 after glucose starvation for 0, 1 and 2 h, respectively. (H) Endogenous RNA pull‐down assays showed the interaction between PKM2 and VAL after glucose starvation for 0, 1 and 2 h, respectively. Representative images of three independent reproducible experiments are presented (A–E, G and H). Results are presented as mean ± SD of at least three independent experiments. **p < .01

The pyruvate kinase domain (N‐terminal to middle region) of PKM2 had been elucidated as a critical region being responsible for its interaction with Parkin, an E3 ubiquitin ligase of PKM2.^34^, ^35^ Therefore, we conjectured that VAL might be involved in regulating the PKM2–Parkin interaction. The results of RIP assays showed that abundant VAL expression markedly abrogated the PKM2–Parkin interaction; meanwhile, comparing with the control cell, the PKM2–Parkin interaction was strengthened, when VAL was silenced in MGC803 cell (Figure 4A). In parallel, the polyubiquitination level of PKM2 was greatly reduced by VAL overexpression; conversely, silencing of VAL intensively elevated the polyubiquitination level of PKM2 in MGC803 cell (Figure 4B). Moreover, the VAL‐mediated suppression of PKM2 polyubiquitination was only detected in AGS control cell, but not in Parkin‐silenced AGS cell, and silencing of VAL could not further enhance Parkin overexpression‐mediated PKM2 polyubiquitination, supporting the notion that VAL suppresses PKM2 polyubiquitination by disrupting the interaction of PKM2 with Parkin (Figure 4C,D). In addition, the PKM2 protein levels were barely altered in SNU16, AGS, MGC803 and GES‐1 cells with VAL overexpression or suppression, indicating that the polyubiquitination level of PKM2 that was regulated by VAL does not affect the protein stability of PKM2 (Figure 4E).

Interestingly, in response to glucose starvation, the expression level of VAL was gradually elevated over time in AGS cell (Figure 4F), simultaneously the PKM2–Parkin interaction and the polyubiquitination level of PKM2 were markedly decreased in a time‐dependent manner (Figure 4G). In addition, gradually increased PKM2 protein was enriched by the endogenous VAL, when AGS cell was subjected to glucose starvation (Figure 4H), further indicating that the interaction between VAL and PKM2 was greatly enhanced in GC cells to resist the nutrient‐deficient conditions. Collectively, the upper mentioned results clearly demonstrated that VAL binds with PKM2 to abrogate the interaction between PKM2 and Parkin, resulting in suppression of the PKM2 polyubiquitination in GC cells.

### 3.5 VAL promotes PKM2‐mediated glycolysis in gastric cancer

**FIGURE 5:**
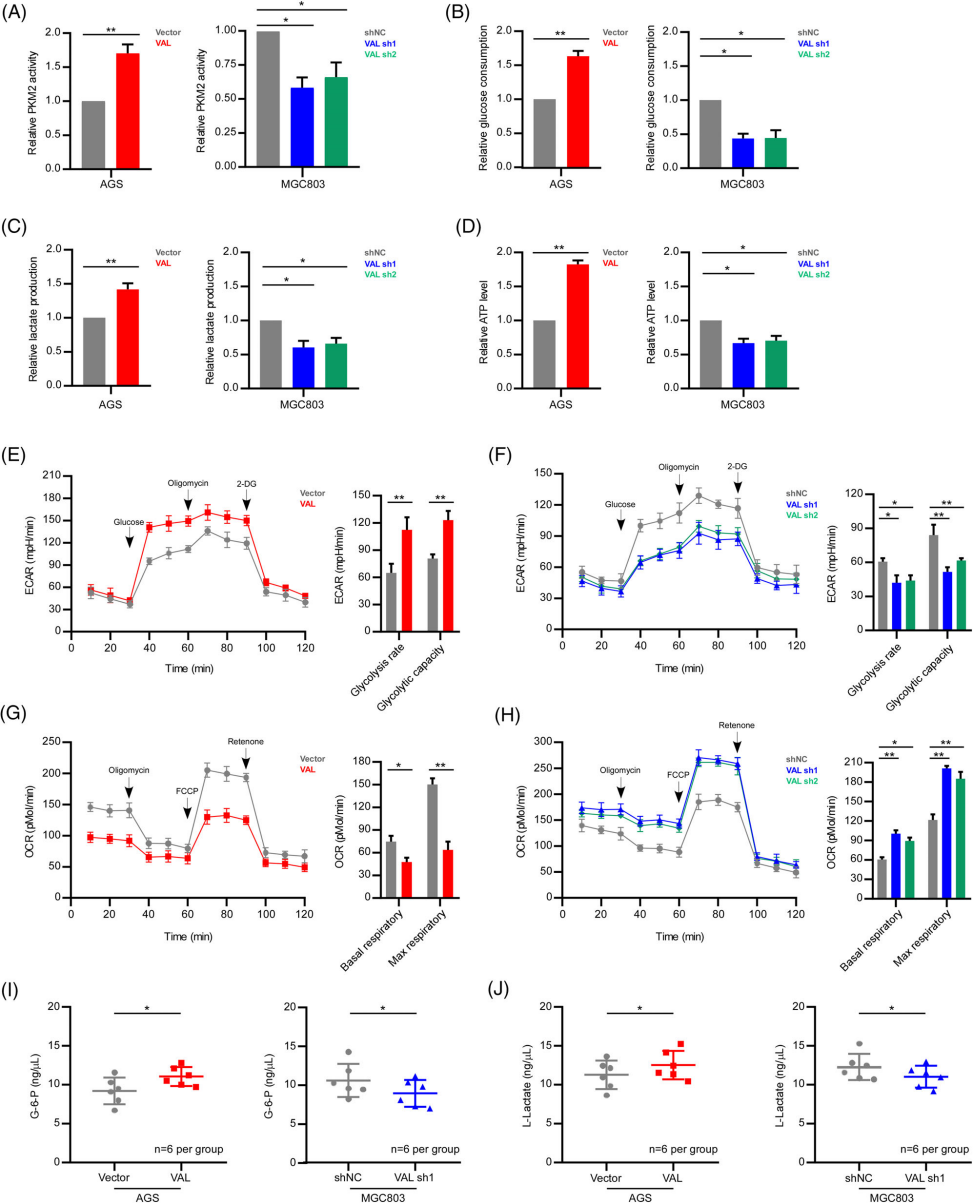
VAL promotes PKM2‐mediated glycolysis in gastric cancer. (A) Pyruvate kinase activity assays were conducted in the indicated cells transfected with Flag‐PKM2 plasmid. (B–D) Glucose consumption (B), lactate production (C) and ATP levels (D) of the indicated cells were determined under hypoxia condition (1% O_2_). (E and F) Analysis of ECAR in the indicated AGS (E) and MGC803 (F) cells under hypoxia condition (1% O_2_). ECAR after glucose injection indicates the glycolysis rate. ECAR after oligomycin injection indicates glycolytic capacity. (G and H) Analysis of OCR in the indicated AGS (G) and MGC803 (H) cells under hypoxia condition (1% O_2_). OCR before oligomycin injection indicates basal respiratory rate. OCR after FCCP (carbonyl cyanide *p*‐[trifluoromethoxy]‐phenyl‐hydrazone) injection indicates the maximum respiratory rate. (I and J) G‐6‐P (I) and L‐lactate (J) in the serum of nude mice inoculated the indicated cells were detected. Results are presented as mean ± SD of at least three independent experiments. *p < .05, **p < .01

Subsequently, we explored the biological function of VAL as a suppressor of PKM2 polyubiquitination. Flag‐tagged PKM2 protein, purified by immunoprecipitation assay, was used to measure the pyruvate kinase activity of PKM2, which showed that the enzymatic activity of PKM2 was significantly elevated when VAL was overexpressed in AGS and SNU16 cells, meanwhile silencing of VAL obtained an opposite result in MGC803 cells (Figure 5A and Figure S5A). Additionally, in cells with silenced Parkin expression, the enzymatic activity of PKM2 was greatly elevated, and overexpression of Parkin markedly inhibited PKM2 activity; however, overexpression or suppression of VAL could not further alter PKM2 enzymatic activity, suggesting that VAL regulates PKM2 activity in a Parkin‐dependent manner (Figure S6A–D).

Due to the pivotal role of PKM2 in glycolysis, subsequently, we investigated the impact of VAL on anaerobic glycolysis and oxidative phosphorylation. The results revealed that the glucose consumption, lactate production and ATP levels were markedly enhanced in AGS and SNU16 cells with VAL overexpression, while these effects were largely diminished by silencing of VAL in MGC803 cells (Figure 5B–D and Figure S5B–D). Furthermore, the ECAR and OCR of indicated cells were measured to validate the influence of VAL on metabolism. The glycolytic capacity and rate were markedly increased with overexpression of VAL in AGS and SNU16 cells, whereas both rates were restrained by suppression of VAL in MGC803 cells (Figure 5E,F and Figure S5E). Simultaneously, the basal OCR and maximum OCR were greatly reduced by VAL overexpression as compared with vector‐control AGS and SNU16 cells. Conversely, both of the basal and maximal OCR levels were potently elevated in VAL‐silenced MGC803 cells (Figure 5G,H and Figure S5F). Summarily, the results suggest that VAL significantly promotes aerobic glycolysis of GC cells.

Notably, the contents of G‐6‐P and L‐lactate in serum, which are the glycolysis‐related organic acids, were also upregulated in mice bearing tumours formed by VAL‐overexpressed AGS cells as compared to those with vector‐control AGS cells; meanwhile, the G‐6‐P and L‐lactate levels were diminished in those bearing xenografts formed by VAL‐silenced MGC803 cells as compared to those with scramble‐control cells (Figure 5I,J). Summarily, our results prove that VAL promotes the enzymatic activity of PKM2 and enhances aerobic glycolysis of GC cells.

### 3.6 PKM2 is essential for VAL‐induced malignant progression and glycolysis of gastric cancer cells

**FIGURE 6:**
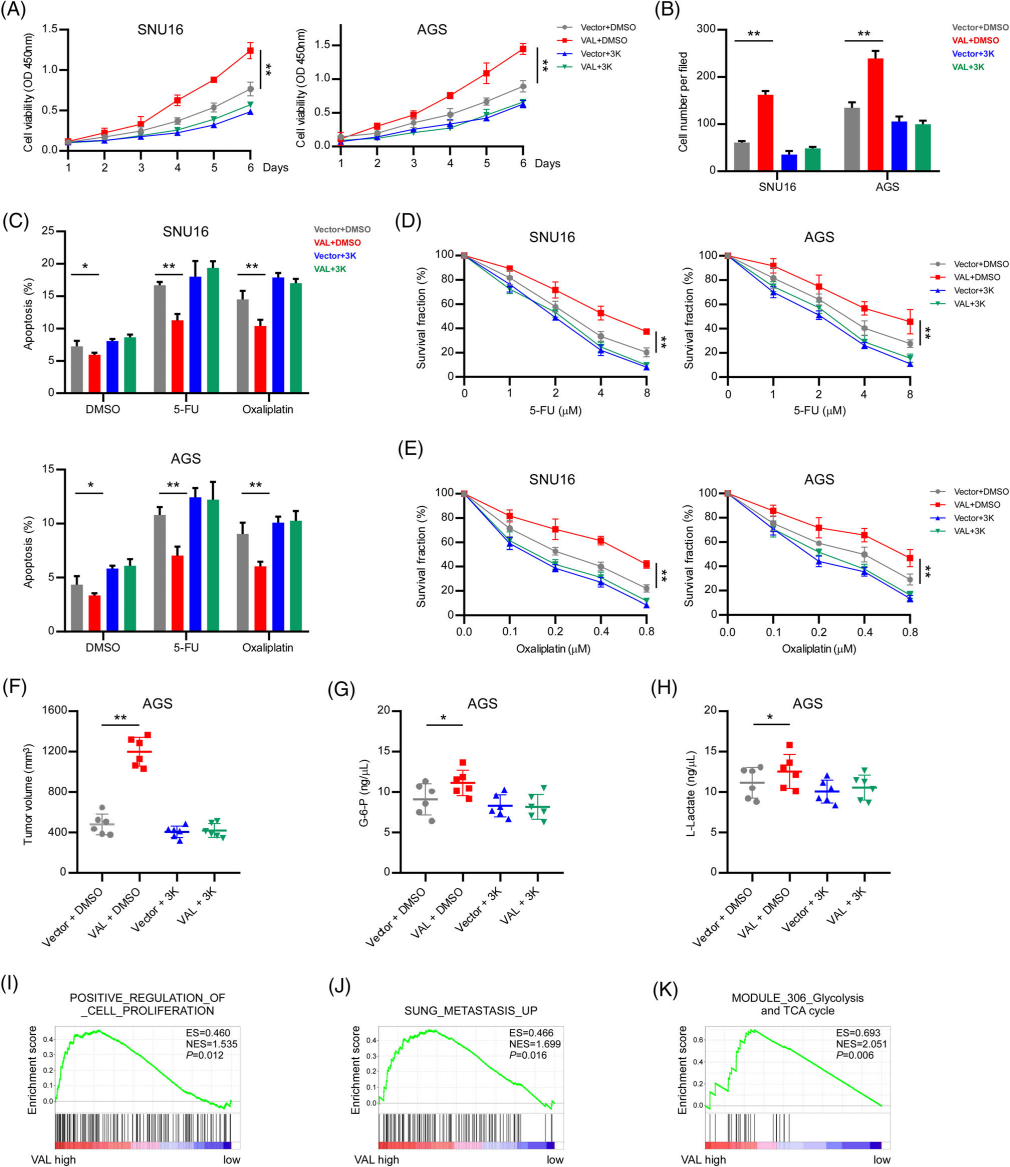
PKM2 is essential for VAL‐induced malignant progression and glycolysis of gastric cancer cells. (A) CCK8 assays were conducted in the indicated cells. (B) Quantification of the indicated cells cultured for 24 h on Matrigel‐coated plates (five random fields of view per well were counted). (C) Apoptosis rates were measured by FACS analysis in the indicated cells treated with 10 μM 5‐FU or 5 μM oxaliplatin for 24 h. (D and E) CCK8 assays were conducted in the indicated cells treated with 0.3 μM Compound 3K and the indicated concentrations of 5‐FU (D) or oxaliplatin (E). (F) The volume of subcutaneous xenograft dissected from nude mice inoculated the indicated cells in each group. (G and H) G‐6‐P (G) and L‐lactate (H) in the serum of nude mice inoculated the indicated cells were detected. (I–K) Gene set enrichment analysis (GSEA) of the TCGA STAD datasets shows positive correlation between higher VAL expression with gene signatures during cell proliferation, metastasis and glycolysis. Results are presented as mean ± SD of at least three independent experiments. *p < .05, **p < .01

To determine the functional significance of PKM2 in VAL‐induced GC phenotype, Compound 3K, a PKM2‐specific inhibitor, was applied in the following assays. As shown in cellular functional assays, VAL‐induced enhancement of cell proliferation, invasion and chemoresistance abilities were significantly abrogated in Compound 3K treatment groups, which were maintained in DMSO treatment groups (Figure 6A–E). Of note, the malignant phenotypes of both VAL‐overexpressed and vector‐control cells treated with Compound 3K were suppressed to a similar lower levels, compared to cells treated with DMSO (Figure 6A–E).

In vivo experiments were further performed. As shown in Figure 6F, the volumes of tumours formed by VAL‐overexpressed AGS cells were significantly increased in DMSO treatment groups, whereas the tumour promotion effect of VAL was abolished by Compound 3K (Figure 6F). As for the effects of VAL on glycolysis, the VAL‐induced elevation of G‐6‐P and L‐lactate levels in serums were markedly suppressed by Compound 3K, which were maintained under DMSO treatment. Notably, after mice were treated with Compound 3K, the G‐6‐P and L‐lactate levels in serums markedly decreased compared to those treated with DMSO (Figure 6G,H), suggesting that VAL promotes malignant progression and glycolysis of GC in a PKM2‐dependent manner. Moreover, the gene set enrichment analysis (GSEA) in TCGA STAD dataset illustrated that VAL level was not only positively associated with upregulated gene signatures of cancer biology processes, including cell proliferation and metastasis (Figure 6I,J), but also significantly associated with the gene signature of glycolysis in GC tissues (Figure 6K), which further supported our notion that higher VAL expression was related with cancer malignant progression and glycolysis in GC patients. Taken together, our findings suggest that VAL accelerates GC malignant progression via strengthening PKM2 enzymatic activity.

## 4 DISCUSSION

Cellular metabolism is critical for the status and function of cells. It has been well established that metabolic reprogramming of cancer cell presents as one of the hallmarks of cancer. Notably, for cancer cells, even in the presence of abundant oxygen, aerobic glycolysis, a phenomenon termed ‘the Warburg effect’ predominantly occurred in cancer cells, which may enhance anabolic metabolism by providing plenty of biomass for macromolecular biosynthesis during cancer cell growth and proliferation.^36^ Upregulated protein expression level or enzymatic activity of GLUT1, HK2, PFK and PKM2 have been well documented in many human tumour types; in this light, the positron emission tomography (PET) is generally used to detect ^18^F‐fluorodeoxyglucose (^18^F‐FDG) uptake for cancer diagnosis in current clinical applications.^6^ Whereas, the underlying mechanisms mediating dysregulation of the core enzymes of aerobic glycolysis in GC remain largely unknown.

Recently, accumulated studies have elucidated that ubiquitination/deubiquitination post‐translational modification system functions as an important approach to regulate metabolic reprogramming and metabolic enzymes in diverse cancer types.^37^, ^38^, ^39^ Despite E3 ligases, like FBXW7, Malin and Nedd4,^40^, ^41^, ^42^ and deubiquitinase, like PSMD14,^43^ are involved in regulating ubiquitination and proteasome‐dependent degradation of PKM2, the E3 ligase Parkin‐mediated PKM2 ubiquitination markedly abrogates the enzymatic activity of PKM2 without an effect on its protein stability, which may be reversed by deubiquitinase OTUB2,^34^, ^35^ suggesting that the regulatory mechanism on PKM2 enzymatic activity and expression level by ubiquitination is complex and largely context‐dependent. Therefore, the core factors being critical for modulating the biological function of PKM2 in GCs remain to be explored.

In the current study, we elucidated that our previously identified vimentin‐associated lncRNA, VAL, presents as a novel binding partner of the glycolytic core enzyme PKM2 in cytoplasm of GC cells. Interestingly, VAL overexpression barely influences the protein level of PKM2, but potently enhances enzymatic activity of PKM2 in glycolysis process of GC cells. Furthermore, we found that VAL binds the pyruvate kinase domain (N‐terminal to middle region) of PKM2, which is the region of PKM2 interacting with the E3 ligase Parkin as previously reported. Subsequently, we proved that upregulated VAL competitively binds to PKM2 and abrogates the interaction of Parkin with PKM2 in a dose‐dependent manner, therefore inhibiting Parkin‐mediated polyubiquitination and causing enzymatic activity suppression of PKM2 in GC cells. Interestingly, we noticed that pyruvate carboxylase (PC), an enzyme converts the pyruvate to oxaloacetate in the first bypass reaction of gluconeogenesis, and has also been detected in the elution of VAL RNA pull‐down assay (Figure 3A). In addition, our preliminary assays indicated that ectopic overexpressed VAL moderately suppresses PC activity, hinting that VAL may promote GC malignant progression by enhancing PKM2 activity and diminishing PC activity synergistically. Moreover, we initially found that VAL barely regulates PKM2 phosphorylation on Ser‐37 and Tyr‐105, as well as oligomerization of PKM2 (dimer and tetramer), which are critical for enzymatic activity of PKM2.^44^, ^45^ These results illustrate an lncRNA‐dependent mechanism underlying provocation of glycolysis via regulating post‐translational modification of PKM2 in GC, suggesting that the regulatory mechanisms of glycolysis in cancers are sophisticated, which need to be further explored and understood.

The critical role of lncRNA VAL has been deciphered in LAD, which is transcriptionally upregulated by AKT activation and stabilizes vimentin protein to promote systemic metastasis of LAD. Moreover, the distinct potency of VAL as a therapeutic target and prognosis marker for LAD has been validated by multiple assays.^33^ Importantly, artificially designed and chemosynthetic molecules, such as siRNA, ASO (antisense oligonucleotides) and Morpholinos, are potentially effective drugs for targeting treatment of cancers.^46^ In light of the aforementioned data, we further elucidated that the VAL level is elevated in GCs and is associated with malignant progression and poor prognosis of GC patients. In addition, our results proved that upregulated VAL expression leads to enhancing glucose consumption, lactate production and cellular ATP production through enhancing PKM2 activity, further resulting in promotion of GC malignant phenotypes, including proliferation, invasion and chemoresistance. Notably, glucose starvation enhances VAL expression, suggesting that VAL plays a pivotal role in GC cell to resist nutrients deficiency and metabolic reprogramming, further presents as a promising prognosis marker and therapeutic target for GCs.

## 5 CONCLUSIONS

In summary, our current study illustrates that VAL is an important regulator of PKM2‐dependent glycolysis process and also functions as a propellant of GC progression. Therefore, our findings strengthen our knowledge on the regulatory mechanism of PKM2 enzymatic activity by Parkin‐mediated polyubiquitination and the biological function of VAL in various cancer‐related contexts, which further provide a foundation for targeting VAL or PKM2 as potential biomarkers in GC diagnosis and treatment.

## CONFLICT OF INTEREST

The authors declare that there is no conflict of interest.

## Supporting information

Figures S1‐S6

Tables S1‐S2

## Data Availability

The data that support the findings of this study are available from the corresponding author upon reasonable request.
