# Supplementary material for: Long non‐coding RNA VAL facilitates PKM2 enzymatic activity to promote glycolysis and malignancy of gastric cancer
Source: Clin Transl Med. 2022 Oct 13;12(10):e1088. doi: 10.1002/ctm2.1088 (PMC9561166; doi:10.1002/ctm2.1088)
Supplement: Supplementary file 1 — Figures S1‐S6 [file CTM2-12-e1088-s001.docx]

**Supplementary Figures**


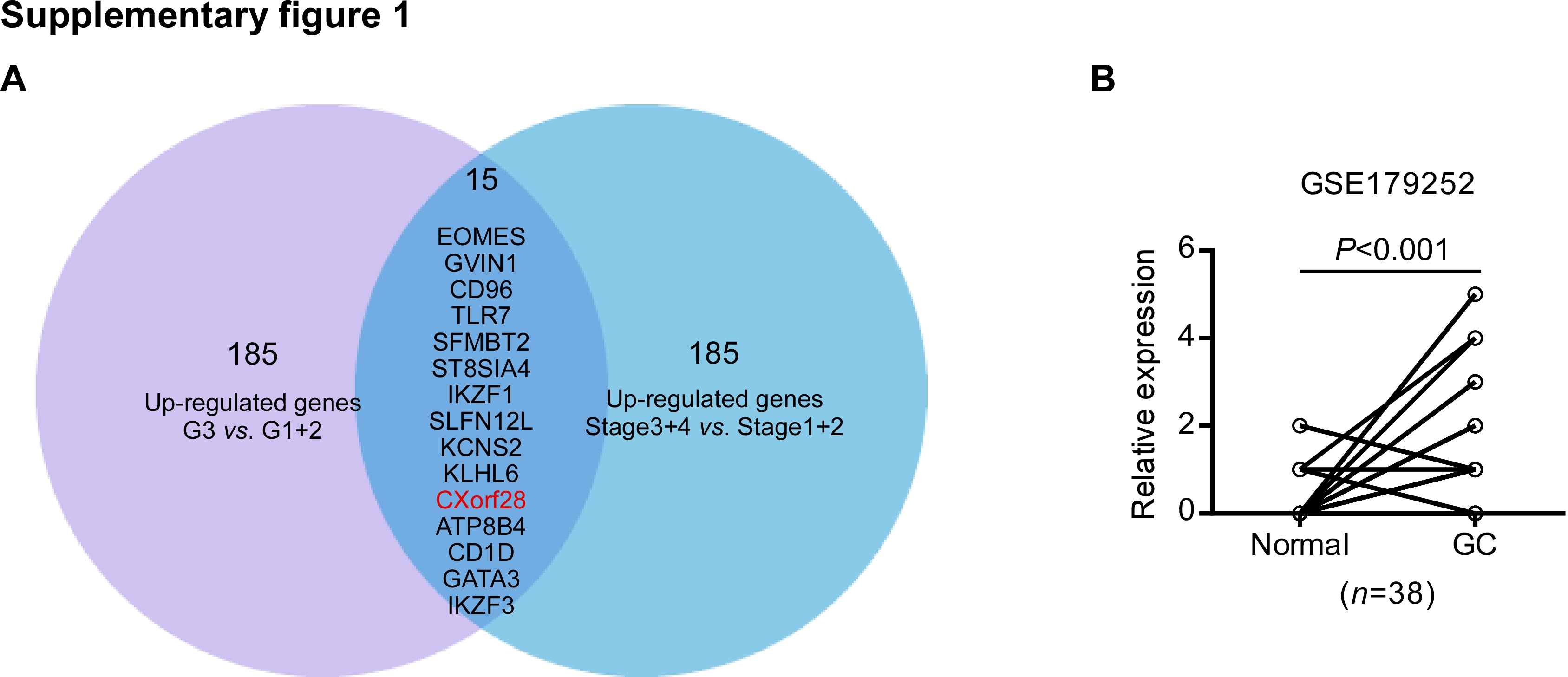


**Supplementary figure 1.** (A) Venn diagram showed up-regulated genes in comparison between G3 and G1+2, and Stage 3+4 and Stage 1+2 of TCGA STAD dataset. (B) VAL expression in the GSE179252 Chinese GC cohort.


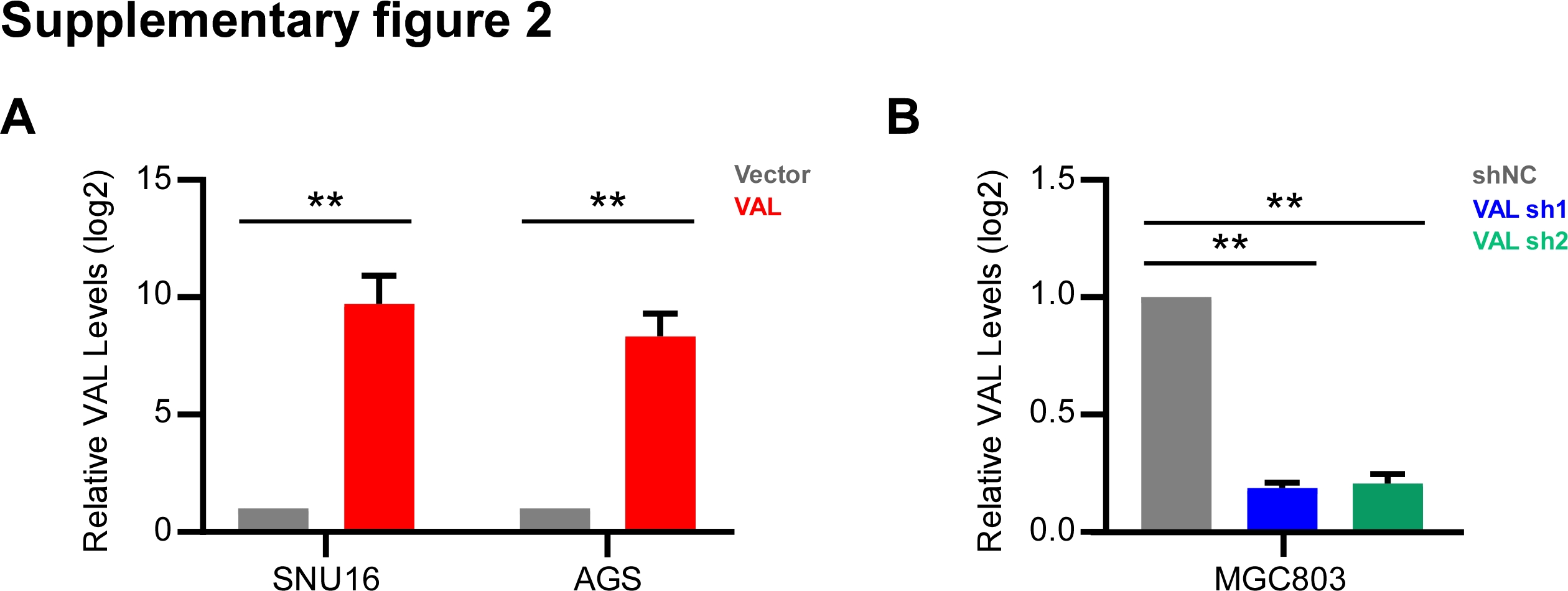


**Supplementary figure 2.** (A), (B) qRT-PCR analysis were employed to validate the stable cell lines with VAL overexpression (A) or VAL expression silenced by shRNAs (B). Results are presented as mean ± SD of three independent experiments. ***P* < 0.01.


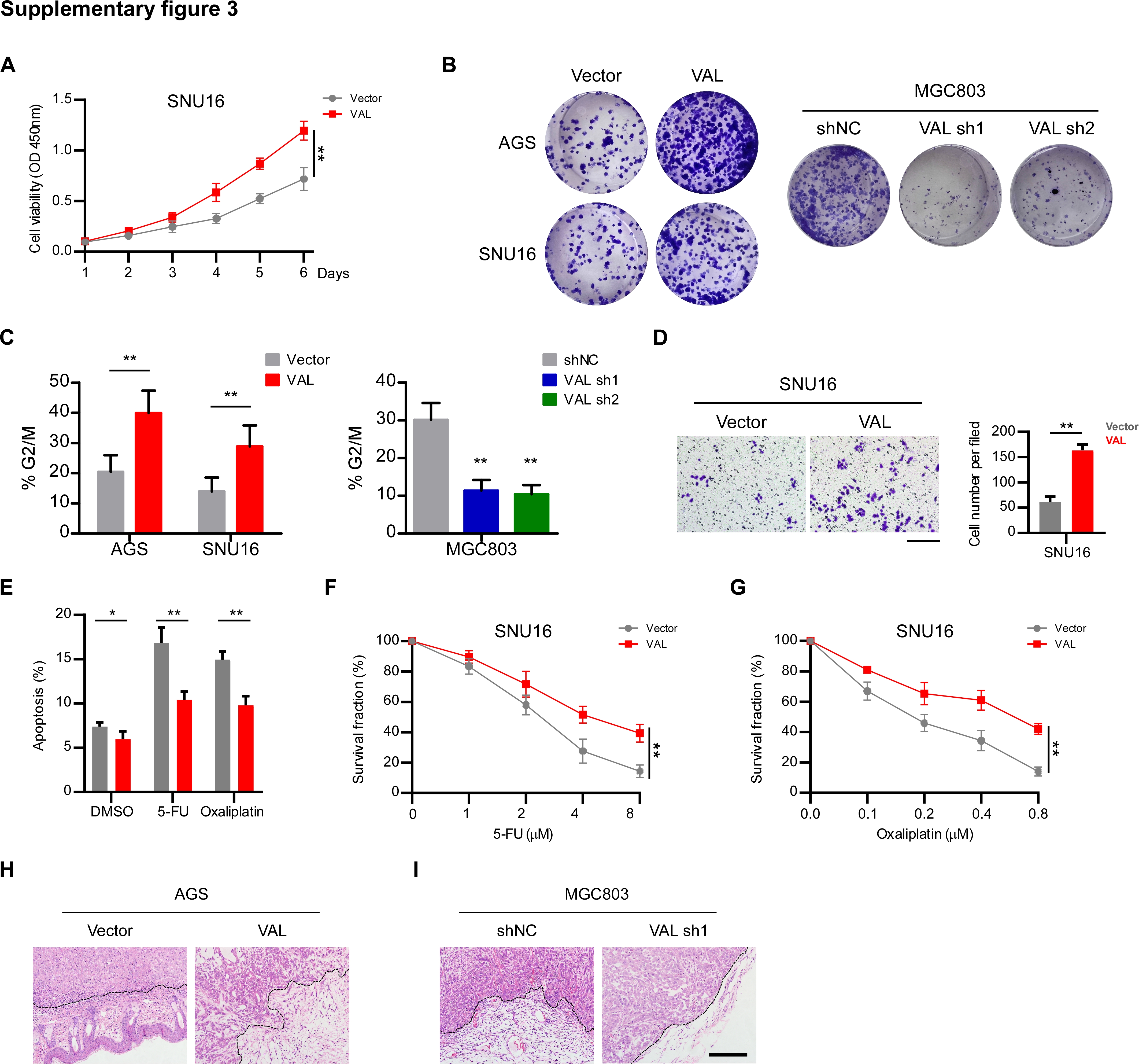


**Supplementary figure 3.** (A) CCK8 assays were conducted in the indicated cells. (B) Colony formation assays were performed with indicated cells. (C) Percentage of cells in G2/M phase of mitosis. (D) Representative images (left) and quantification (right) of the indicated cells cultured for 24 h on Matrigel-coated plates (five random fields of view per well, scale bar: 100 μm). (E) Apoptosis rates were measured by FACS analysis in the indicated cells treated with 10 μM 5-FU or 5 μM oxaliplatin for 24 h. (F), (G) CCK8 assays were conducted in the indicated cells treated with 0.3 μM Compound 3K and the indicated concentrations of 5-FU (F) or oxaliplatin (G). (H), (I) H&E staining of both the dermal tissue and tumor tissue in the subcutaneous tumors xenografted with the indicated cells (scale bar: 100 μm). Results are presented as mean ± SD of at least three independent experiments. **P* < 0.05, ***P* < 0.01.


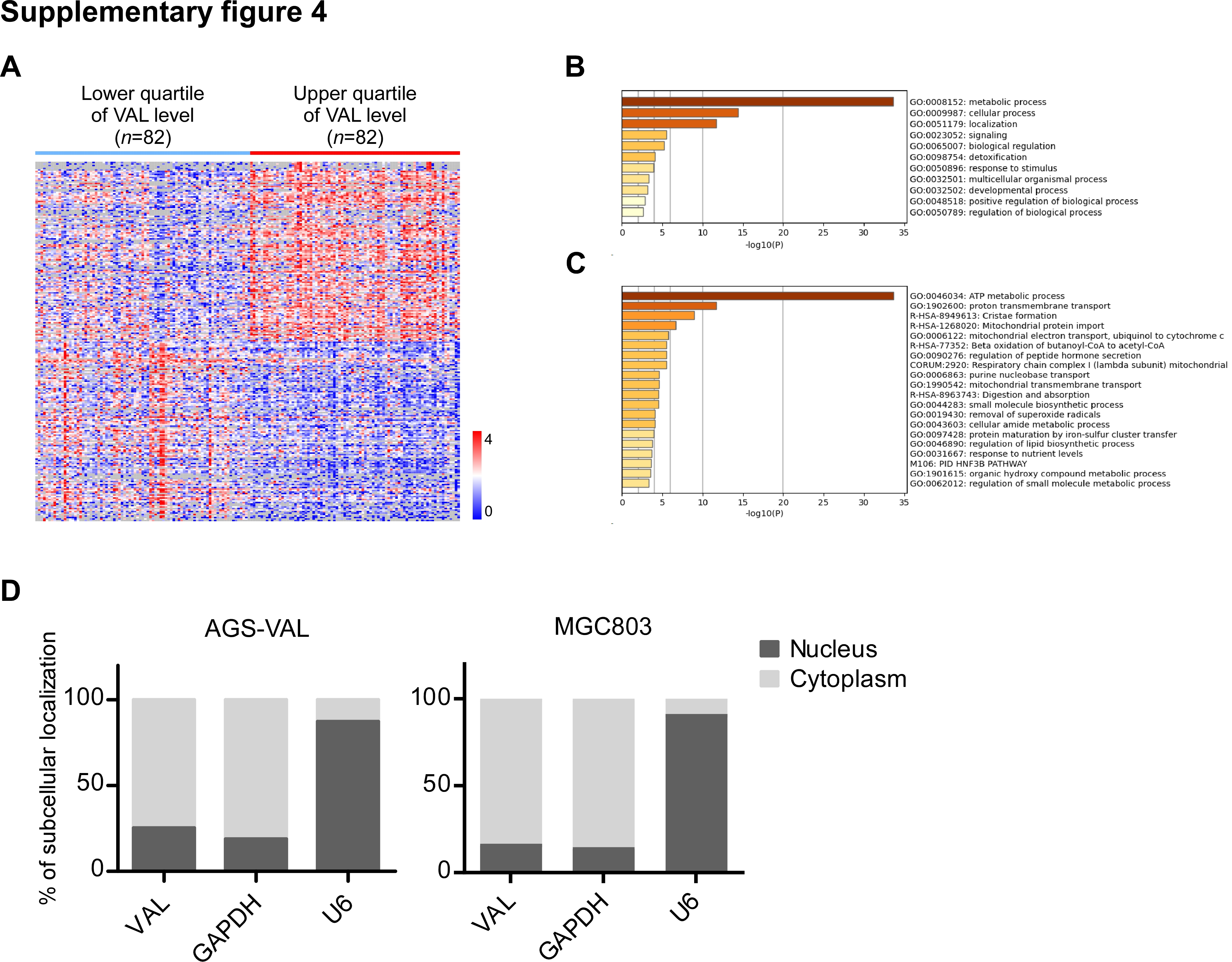


**Supplementary figure 4.** (A) Heatmap showed the significantly altered genes in comparison between the subgroups with upper quartile or lower quartile levels of VAL in TCGA STAD dataset. (B), (C) Biological processes associated with up-regulated genes in patients with upper quartile VAL levels were identified by the Metascape program (metascape.org). (D) qRT-PCR results showed the subcellular localization of VAL in indicated cells.


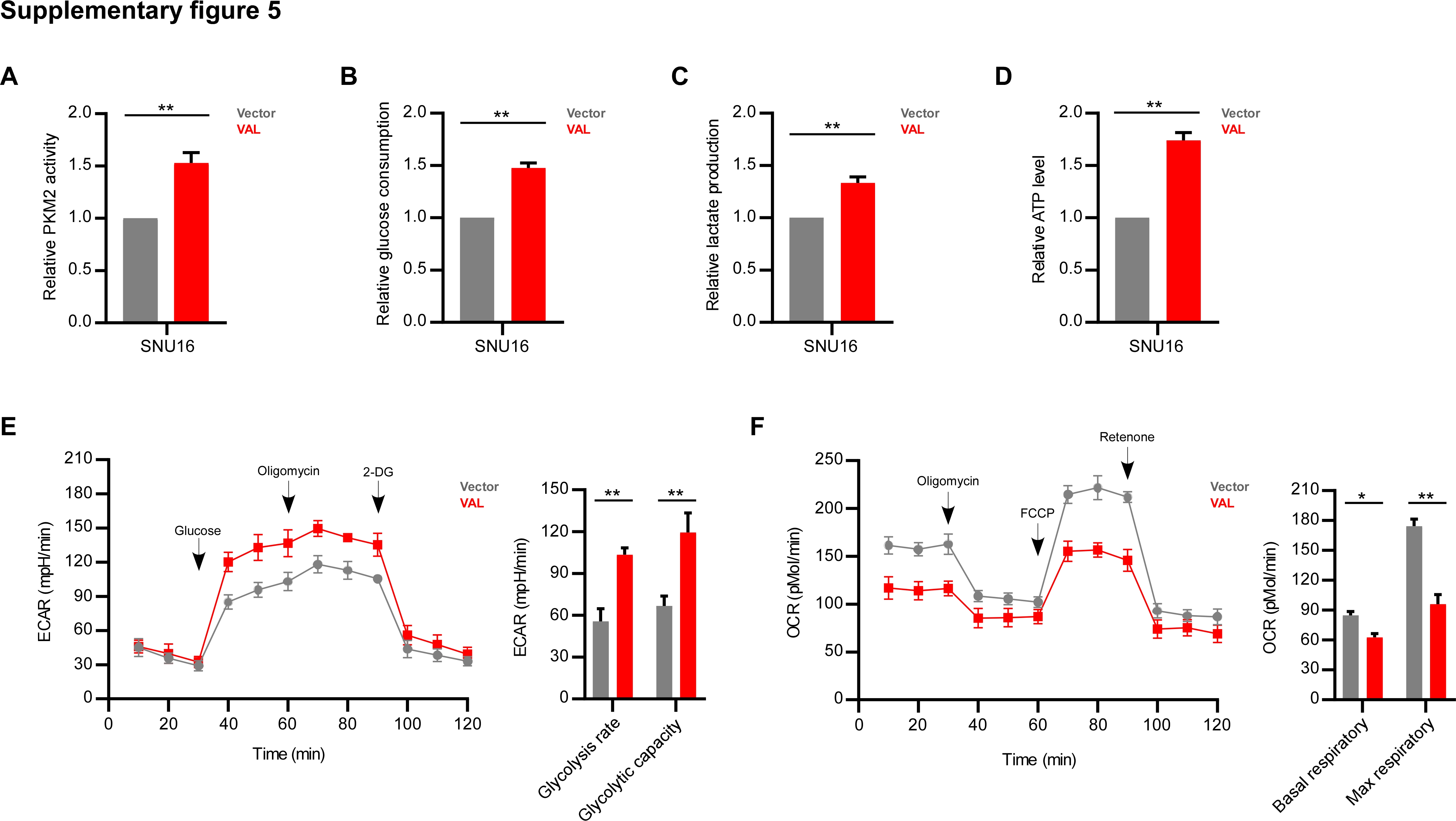


**Supplementary figure 5.** (A) Pyruvate kinase activity assays were conducted in the indicated SNU16 cells transfected with Flag-PKM2 plasmid. (B)-(D) Glucose consumption (B), lactate production (C) and ATP levels (D) of the indicated cells were determined. (E) Analysis of ECAR in the indicated SNU16 cells. ECAR after glucose injection indicates the glycolysis rate. ECAR after oligomycin injection indicates glycolytic capacity. (F) Analysis of OCR in the indicated SUN16 cells. OCR before oligomycin injection indicates basal respiratory rate. OCR after FCCP (carbonyl cyanide p-[trifluoromethoxy]-phenyl-hydrazone) injection indicates the maximum respiratory rate. Results are presented as mean ± SD of at least three independent experiments. **P* < 0.05, ***P* < 0.01.


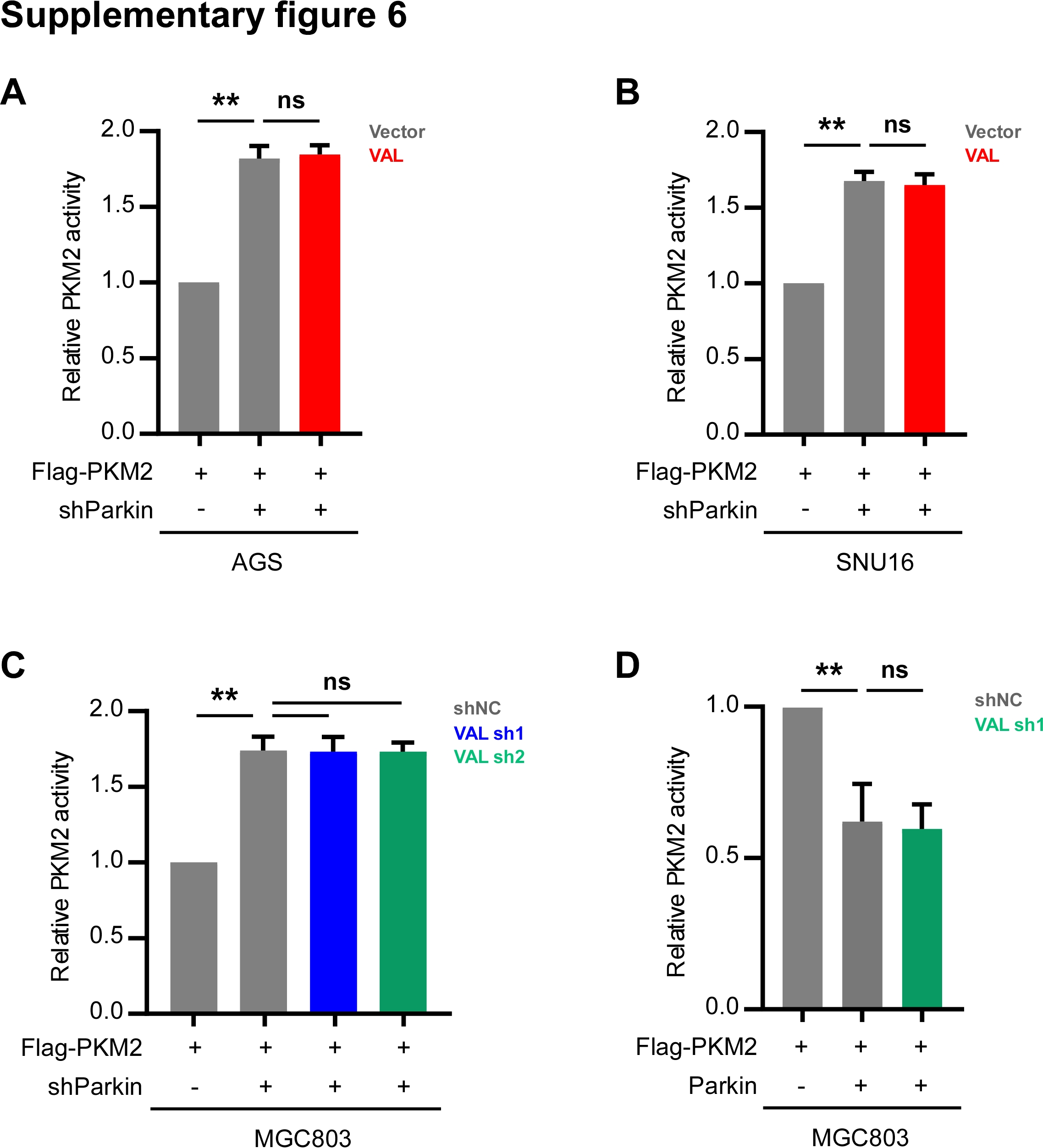


**Supplementary figure 6.** (A)-(D) Pyruvate kinase activity assays were conducted in the indicated AGS (A), SNU16 (B) and MGC803 (C), (D) cells transfected with the indicated plasmids. Results are presented as mean ± SD of at least three independent experiments.
